# Self-referenced interferometry for single-shot detection of vector-vortex beams

**DOI:** 10.1038/s41598-022-21485-w

**Published:** 2022-10-14

**Authors:** Praveen Kumar, Naveen K. Nishchal, Takashige Omatsu, A. Srinivasa Rao

**Affiliations:** 1 Graduate School of Science and Engineering, Chiba University, Chiba, 263-8522 Japan; 2 Molecular Chirality Research Center, Chiba University, Chiba, 263-8522 Japan; 3 Department of Physics, Indian Institute of Technology Patna, Bihta, Patna, Bihar 801 106 India

**Keywords:** Optics and photonics, Physics

## Abstract

Vector-vortex (VV) beams are of significant interest for various applications. There have been substantial efforts toward developing a fast and efficient method for the characterization of generated VV beams which is crucial for their usage. Polarimetric approaches are commonly used to identify unknown VV beams but require multiple intensity recordings. This paper demonstrates a technique to detect VV beams and identify their parameters using the concept of self-referenced interferometry. The approach uses a single recorded interferogram to determine the beam parameters that allow rapid detection. The method even enables detection of VV beams having high-order optical vortices.

## Introduction

Advancements in technology for light shaping are responsible for opening new opportunities for optical systems. Intensity, phase and polarization are the parameters of light that can be shaped spatially and temporally for a purpose^1^. Such optical fields are termed structured light or tailored light. Structured laser beams with complex wavefront structures are gradually occupying prominent positions in many optical technologies^2–4^. Efforts on light shaping have uncovered new features of light, such as the formation of optical vortices caused by helical wavefront^2^. Vortex beams hold phase singularity where phase remains indeterminate. Such beams having non-uniform polarization are referred to as vector-vortex (VV) beams. These beams consist of both phase and V-point polarization singularities and are known as cylindrical-vector beams^5^. VV beams can be described using higher-order Poincaré sphere^6^.

Commonly known VV beams are the azimuthally and radially polarized beams. These beams exhibit unique focusing properties and have found applications in imaging and microscopy^7–9^. Vortex beams carry orbital angular momentum (OAM) arising from the azimuthal phase dependence^10–12^. Besides, light can also carry spin angular momentum associated with circular polarization^10^. For this reason, VV beams have found attractive applications in optical manipulation and material processing^12–15^. Topological charge (TC) is a parameter that denotes the order of singularity and the associated OAM mode. Vortex beams can be represented by Laguerre–Gaussian (LG) modes, which can have many orthogonal states characterized by integral values of azimuthal index or the TC. In contrast, polarization offers only two orthogonal states. Large degrees of freedom brought by this feature of vortex beams and VV beams have made them very attractive for applications in optical communications and cryptography^16–20^. Furthermore, VV beams play a crucial role in studying light-matter interactions, spin–orbit interactions, and quantum optics^11,21–24^. Recent developments with the fundamental theories and applications of VV beam in diverse areas of classical and quantum optics have been reported in review articles^1–3,25–27^.

Recently, several technologies for the generation of structured beam have been developed, such as q-plate, spatial light modulator (SLM), metasurfaces, etc^28–35^. Efficient technique for the characterization of VV beams is also a prerequisite for its emerging innovations and applications. Therefore, efforts have been made to develop fast and efficient methods for the detection of structured beams^36–41^. Methods based on diffractive and interferometric approaches have been reported for the TC measurement of the optical vortices^42–46^. Previous works have shown the use of self-referenced interferometry for the detection of scalar vortices^47,48^. Many other approaches have also been reported for the analysis of phase and polarization singularities^49–51^. Usually, a polarimetric method is used for the detection of VV beams^52–55^. In this approach, Stokes parameters of the input beam are measured using at least four intensity recordings. Compact optical set-ups consisting of a digital micro-mirror device have been suggested for the measurement of Stokes parameters for the beam characterization^54^. In a diffraction-based approach, it has been shown that the diffraction patterns formed by VV beams after transmitting through a tilted lens can be analyzed for their detection^56^. In this approach, the diffraction pattern becomes difficult to analyze for high-order VV beams. Another approach is to measure the TC of the orthogonal components of the input VV beam by analyzing a pair of interferograms recorded using a reference beam^57^. Interference-based methods are intuitive and have simpler implementation. However, a standalone reference beam for interference may not always be available while practical applications of VV beams.

In this paper, we demonstrate a method to identify VV beams by estimating parameters through the analysis of a single recorded interferogram obtained without the use of any external reference beam. The concept is based on the principle of self-referenced interferometry. This approach can also identify VV beams consisting of high-order optical vortices efficiently and hence can be widely used in rapid detection of VV beams.

## Principle

Vortex beams can be represented by LG modes which have a helical phase profile. The electric field amplitude of a LG beam propagating along the *z*-direction, can be expressed as^2^,

1 $$\begin{gathered} LG(x,y;\phi ) = \sqrt {\frac{2p\,!}{{\pi \left( {p + \left| {{\kern 1pt} \ell {\kern 1pt} } \right|} \right)\,!}}} \frac{1}{w(z)}\left[ {\frac{r\sqrt 2 }{{w(z)}}} \right]^{{^{{\left| {{\kern 1pt} \ell {\kern 1pt} } \right|}} }} \exp \left[ {\frac{{ - r^{2} }}{{w^{2} (z)}}} \right] \hfill \\ \quad \quad \quad \quad \quad \quad \quad \quad \quad \quad \quad \quad \quad \;L_{p}^{{\left| {{\kern 1pt} \ell {\kern 1pt} } \right|}} \left( {\frac{{2r^{2} }}{{w^{2} (z)}}} \right)\exp \left[ {\frac{{ik_{0} r^{2} z}}{{2\left( {z^{2} + z_{R}^{2} } \right)}} - iG + i\phi } \right]. \hfill \\ \end{gathered}$$

In this equation, $L_{p}^{{\left| {{\kern 1pt} \ell {\kern 1pt} } \right|}}$ denotes the associated Laguerre polynomial with *ℓ* being the azimuthal index, and *p* the radial index. *w*(*z*) is 1/*e* radius of the Gaussian term given by $w(z) = w(0)\left[ {{{\left( {z^{2} + z_{R}^{2} } \right)} \mathord{\left/ {\vphantom {{\left( {z^{2} + z_{R}^{2} } \right)} {z_{R}^{2} }}} \right. \kern-\nulldelimiterspace} {z_{R}^{2} }}} \right]^{1/2}$ with *w*(0) being the beam waist, and *z*_*R*_ the Rayleigh range. *G* denotes the Gouy phase given by $G = \left( {2p + \left| \ell \right| + 1} \right)\tan^{ - 1} \left( {{z \mathord{\left/ {\vphantom {z {z_{R} }}} \right. \kern-\nulldelimiterspace} {z_{R} }}} \right)$, and $r = \sqrt {x^{2} + y^{2} }$ with *x* and *y* are the coordinates in the transverse plane. The term *ϕ* denotes the helical phase distribution.

**Figure 1:**
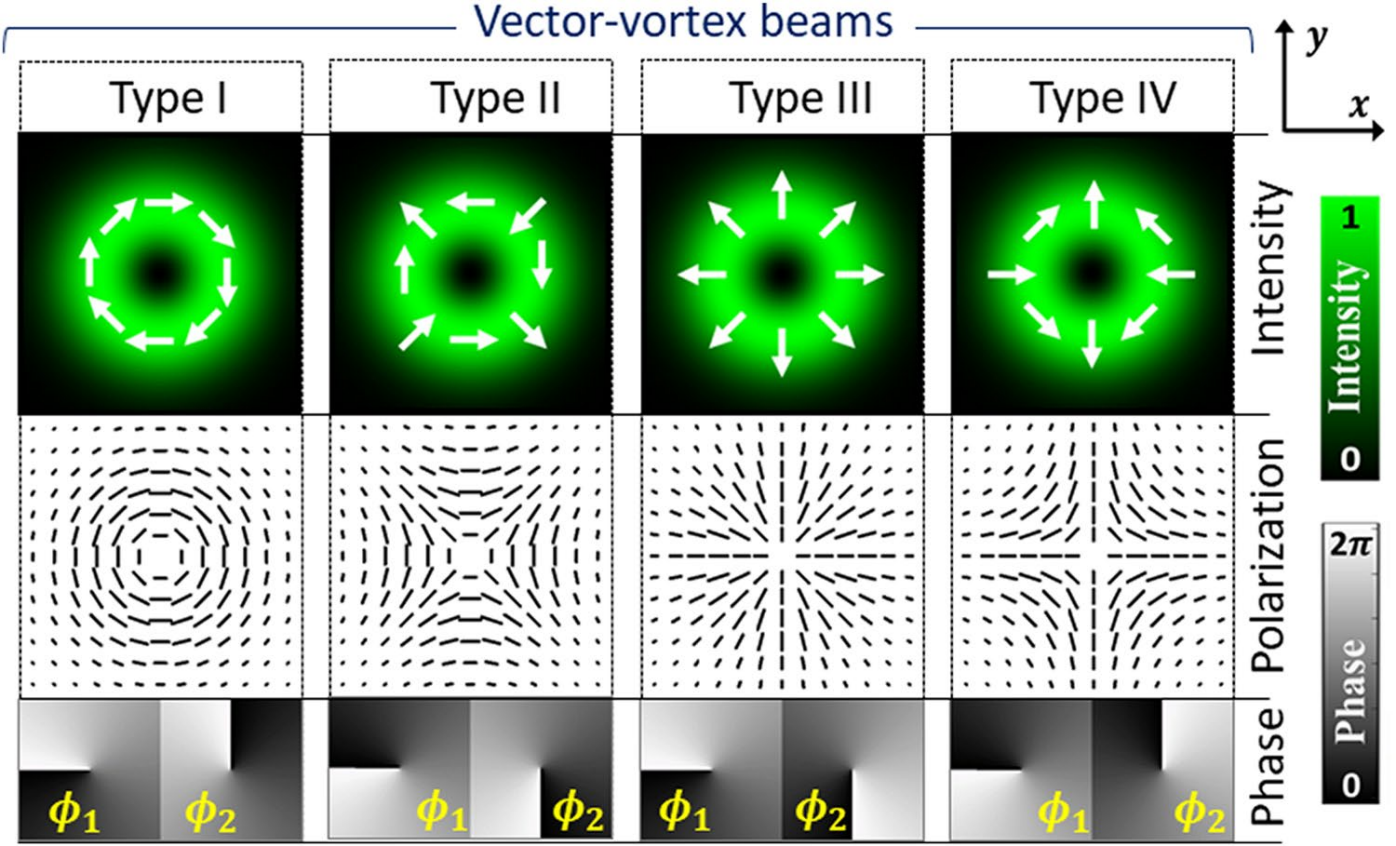
Intensity, polarization and phase distributions of vector-vortex beams of Type-I, II, III and IV for |*ℓ*|= 1.

VV beams can be represented as the superposition of two scalar LG beams in circular polarization basis. The orthogonal electric field components *E*_1_ and *E*_2_ of VV beams can be represented in terms of Jones vector as^35,58^,

2 $$\left[ {\begin{array}{*{20}c} {E_{1}^{{}} (x,y)} \\ {E_{2}^{{}} (x,y)} \\ \end{array} } \right] = \frac{1}{2}\,\left[ {\begin{array}{*{20}c} {1 - i} & { - 1 - i} \\ { - 1 - i} & {1 - i} \\ \end{array} } \right]\;\left[ {\begin{array}{*{20}c} {LG_{1} \left( {x,y;\phi_{1} } \right)} \\ {LG_{2} \left( {x,y;\phi_{2} } \right)} \\ \end{array} } \right]\;,$$

where *ϕ*_1_ and *ϕ*_2_ denote the phase value distributions (PVDs) corresponding to scalar LG modes, denoted as *LG*_1_ and *LG*_2_, respectively. The PVDs for VV beams have azimuthal phase dependence. They are expressed as,

3 $$\begin{gathered} \phi_{1}^{{}} (x,y) = \,\,\ell \,\tan^{ - 1} \left( {y/x} \right) \hfill \\ \phi_{2}^{{}} (x,y) = - {\kern 1pt} \,\ell \,\tan^{ - 1} \left( {y/x} \right) + \delta , \hfill \\ \end{gathered}$$

where the term *δ* is the phase delay and *ℓ* denotes the azimuthal index or the TC. VV beams hold an optical vortex of order |*ℓ*| in each of its polarization component. TC gives the order of phase singularity caused by twist in the optical wavefront. The phase values around singularity change by an integral multiple of 2*π*. Its magnitude is equal to the winding number of the phase per revolution around the singularity, and its sign denotes the direction in which the phase increases (clockwise or anti-clockwise). The most commonly known VV beams are Type-I, II, III and IV^59^. They can be represented by Eq. (2) with an appropriate selection of *ℓ* and *δ*. Their intensity profile, polarization, and PVDs for |*ℓ|*= 1 are illustrated in Fig. 1. For all these beams, the radial index remains zero. Types-I and III are the azimuthally and radially polarized beams, respectively. Types-II and IV are the index inversed polarization distribution of Types-I and III, respectively.

Detection of VV beams using the approach of self-referenced interferometry is discussed. This approach uses off-axis interference between the input VV beam’s separated polarization components. The beam is decomposed into its orthogonal components on a linear polarization basis by transmitting it through a quarter-wave plate (QWP) oriented at − 45º with *x*-axis and a polarizing beam splitter (PBS). Resultant beam components *E*_A_, *E*_B_ are obtained by Jones matrix multiplication of PBS and QWP with the Jones vector of the input beam as follows,

4 $$\begin{gathered} E_{{\text{A}}} (x,y) = \frac{1}{2}\left[ {\begin{array}{*{20}c} 1 & 0 \\ 0 & 0 \\ \end{array} } \right]\;\left[ {\begin{array}{*{20}c} {1 - i} & {1 + i} \\ {1 + i} & {1 - i} \\ \end{array} } \right]\;\left[ {\begin{array}{*{20}c} {E_{1}^{{}} (x,y)} \\ {E_{2}^{{}} (x,y)} \\ \end{array} } \right] \hfill \\ E_{{\text{B}}} (x,y) = \frac{1}{2}\left[ {\begin{array}{*{20}c} 0 & 0 \\ 0 & 1 \\ \end{array} } \right]\;\left[ {\begin{array}{*{20}c} {1 - i} & {1 + i} \\ {1 + i} & {1 - i} \\ \end{array} } \right]\;\left[ {\begin{array}{*{20}c} {E_{1}^{{}} (x,y)} \\ {E_{2}^{{}} (x,y)} \\ \end{array} } \right]. \hfill \\ \end{gathered}$$

The beam component *E*_A_ is linearly polarized along the *x*-axis while the component *E*_B_ is polarized along the *y*-axis. An off-axis interference field is obtained from these beam components using a Mach–Zehnder interferometric set-up. A small amount of tilt is incorporated in the beam component *E*_B_ such that it interferes with the other component at some small angle. Tilt causes small displacement between the interfering beams as they propagate. Therefore, lateral displacement ∆*y* is simultaneously introduced between the interfering beams to ensure their exact overlapping at the imaging plane. The modified beam component *E*^º^_B_ is expressed as,

5 $$E_{{\text{B}}}^{ \circ } = E_{{\text{B}}} (x,y + \Delta y)\exp \left\{ {ik\,(y + \Delta y)\tan \beta } \right\},$$

where *β* denotes the tilt angle applied along the *y*-axis and *k* is the wave number. The beam components *E*_A_ and *E*^º^_B_ propagate a short transmission distance *z* to reach the imaging plane. The resultant field of the beam components is calculated according to Fresnel diffraction solution^60^. Both the beams then pass through a polarizer with a transmission axis oriented at 45º with the *x*-axis. This ensures identical state of polarization of the interfering beams. The intensity distribution of resultant interference field *I*(*x,y*) at the imaging plane is given as,

6 $$I(x,y) = \left| {E_{{\text{A(z)}}} + E_{{\text{B(z)}}}^{ \circ } } \right|^{2} .$$

In this equation, *E*_A(z)_ and *E*^º^_B(z)_ denote the electric field of the interfering beams at the imaging plane where both remain linearly polarized at 45° with the *x*-axis. The resultant intensity distribution, *I*(*x,y*) consists of interference fringes corresponding to the input VV beam. Different VV beams produce a unique pattern in the interferogram that is analysed for their identification.

## Experimental procedure

### Generation of VV beams

**Figure 2:**
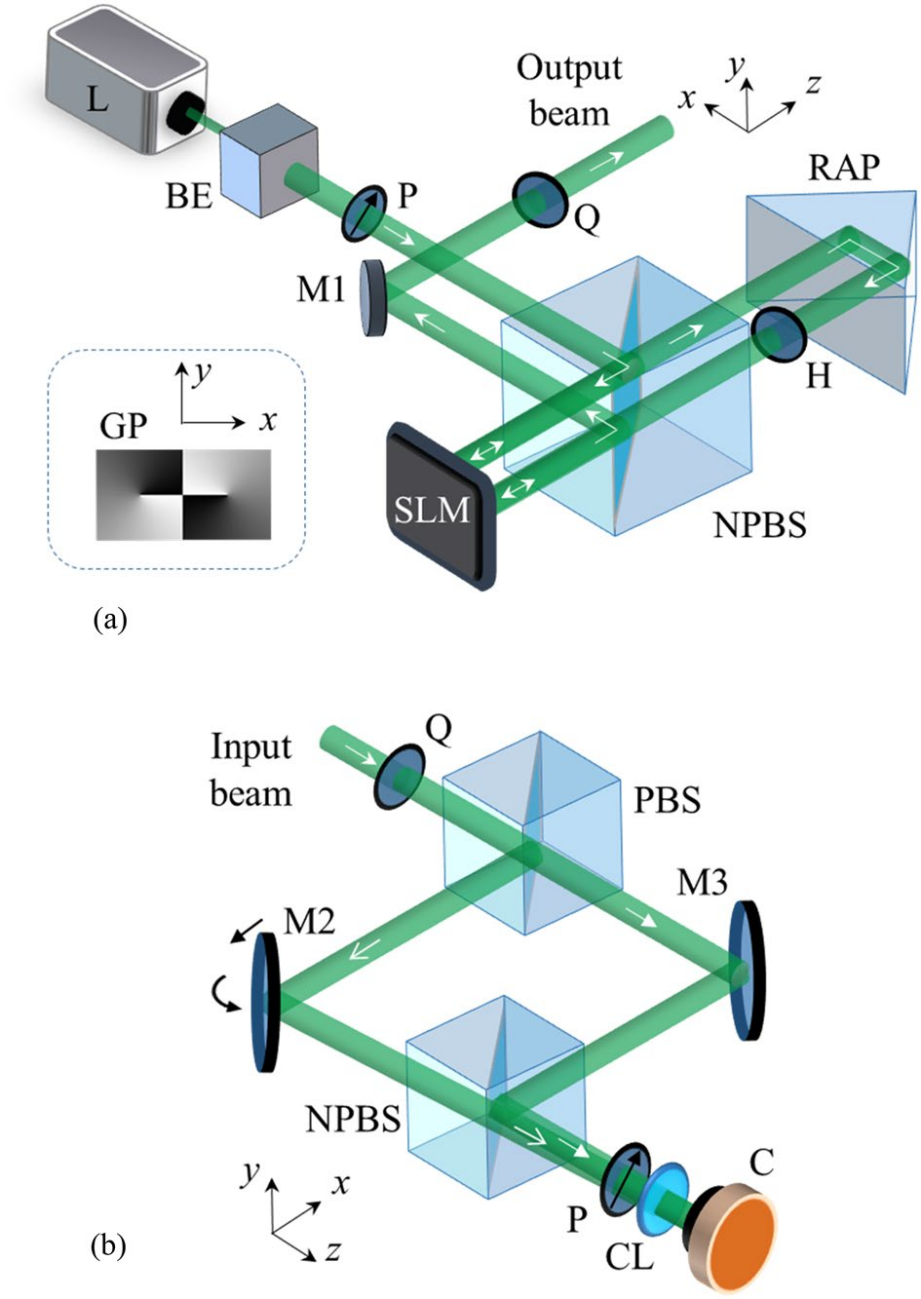
Schematic of the experimental set-up for (**a**) generation and (**b**) detection of VV beams. *BE* beam expander, *P* polarizer, *Q* quarter-wave plate, *H* half-wave plate, *PBS* polarizing beam splitter, *NPBS* non-polarizing beam splitter, *RAP* right-angle prism, *M* mirror, *CL* converging lens, *C* camera, *GP* grayscale pattern displayed on the SLM to generate Type I beam.

The experimental set-up to generate VV beams is shown in Fig. 2a. Laser (Cobolt, Sweden, DPSS laser, 100 mW) is used as a light source to produce a beam of wavelength ~ 532 nm. A reflective-type liquid crystal SLM is utilized for the phase modulation. The SLM (Pluto Holoeye, Germany) has a pixel pitch of 8.0 µm and a resolution of 1920 × 1080 pixels. In this case, the SLM has been utilized to modulate both polarization components of the beam using two grayscale patterns which are displayed side-by-side onto its active area. The grayscale pattern consists of gray levels varying from 0 to 255, corresponding to a phase delay from 0 to 2*π*. The SLM has a total active area of ~ 15.36 × 8.64 mm^2^. Both the grayscale patterns are concatenated such that each pattern utilizes SLM’s active area of ~ 7.68 × 8.64 mm^2^. If the incident beam diameter exceeds this limit, it may cause an error while phase modulation. At the same time, the resolution of phase modulation reduces if the beam diameter is very less. As an optimum solution, the beam diameter is kept within ~ 5 mm using an aperture fixed along with the collimating lens within the beam expander. It can introduce some diffraction effects causing the incident beam to have minor deviations from an ideal Gaussian profile^60^. After collimation, the beam is transmitted through a polarizer to make it linearly polarized along the 45º with the *x*-axis (laboratory horizontal).

The SLM is polarization sensitive and modulates only the light polarized along its slow axis^61^. Its slow axis is kept along the *x*-axis. The collimated laser beam is directed towards the SLM’s display through a non-polarizing beam splitter. After reflection from the half part of SLM’s display, the beam acquires the phase *ϕ*_1_(*x,y*) in one of the beam components. Then the beam is transmitted through a half-wave plate to interchange the vertical and horizontal polarization components. A right-angle prism redirects the beam towards the other part of SLM's display to modulate the phase *ϕ*_2_(*x,y*) in the other component. Finally, the modulated beam is brought to an elliptical polarization basis using a QWP whose slow axis is aligned at 45º with the *x*-axis. The output beam can be represented by Eq. (2). The pattern used for the generation of the Type I beam is shown in Fig. 1a. The incident beam is directed one by one towards the two separate areas of the SLM such that there is no overlapping between them. For correct phase modulation, it is important that the beams are reflected from the designated area of SLM’s display. The alignment is achieved by controlling the tilt and displacement of the right-angle prism along the horizontal and vertical directions. The size and spatial resolution of SLM’s active area limit the maximum TC of the vortex beam that can be generated. In this study, VV beams have been generated that holds an optical vortex of TC up to 20.

### Detection of VV beams

The concept of self-referenced interferometry is utilized for the detection of VV beams, as described in previous section. The configuration of a modified Mach–Zehnder interferometer (MZI) is employed to capture an interferogram, as shown in Fig. 2b. The input VV beam is transmitted through a QWP and a PBS to separate the vertical and horizontal polarization components. These components take different paths of the MZI. Small tilt and lateral displacement are introduced into the beam propagating in one of the arms of MZI. Lateral displacement is applied along the direction of tilt. Mirror M2 is used to control these parameters, as indicated in Fig. 2b. Subsequently, beams are directed towards the imaging plane through a non-polarizing beam splitter. They also transmit through a linear polarizer that makes their polarization state identical. The polarizer’s transmission axis is at 45º with the *x*-axis. The resulting interference field at the imaging plane is recorded by a camera (CMOS Infinity, Lumenera Corp., Canada). The camera has a pixel pitch of 5.2 µm and a resolution of 1280 × 1024 pixels. A converging lens has been used to reduce the beam size to fit into the camera’s active area. The beam components arrive at different angles on the camera for an off-axis interference. The recorded fringe pattern is analyzed for the characterization of the input beam. The alignment is achieved by controlling the tilt and displacement of the mirror M2 while the observation plane (camera) remains fixed just after the MZI.

## Results and analysis

**Figure 3:**
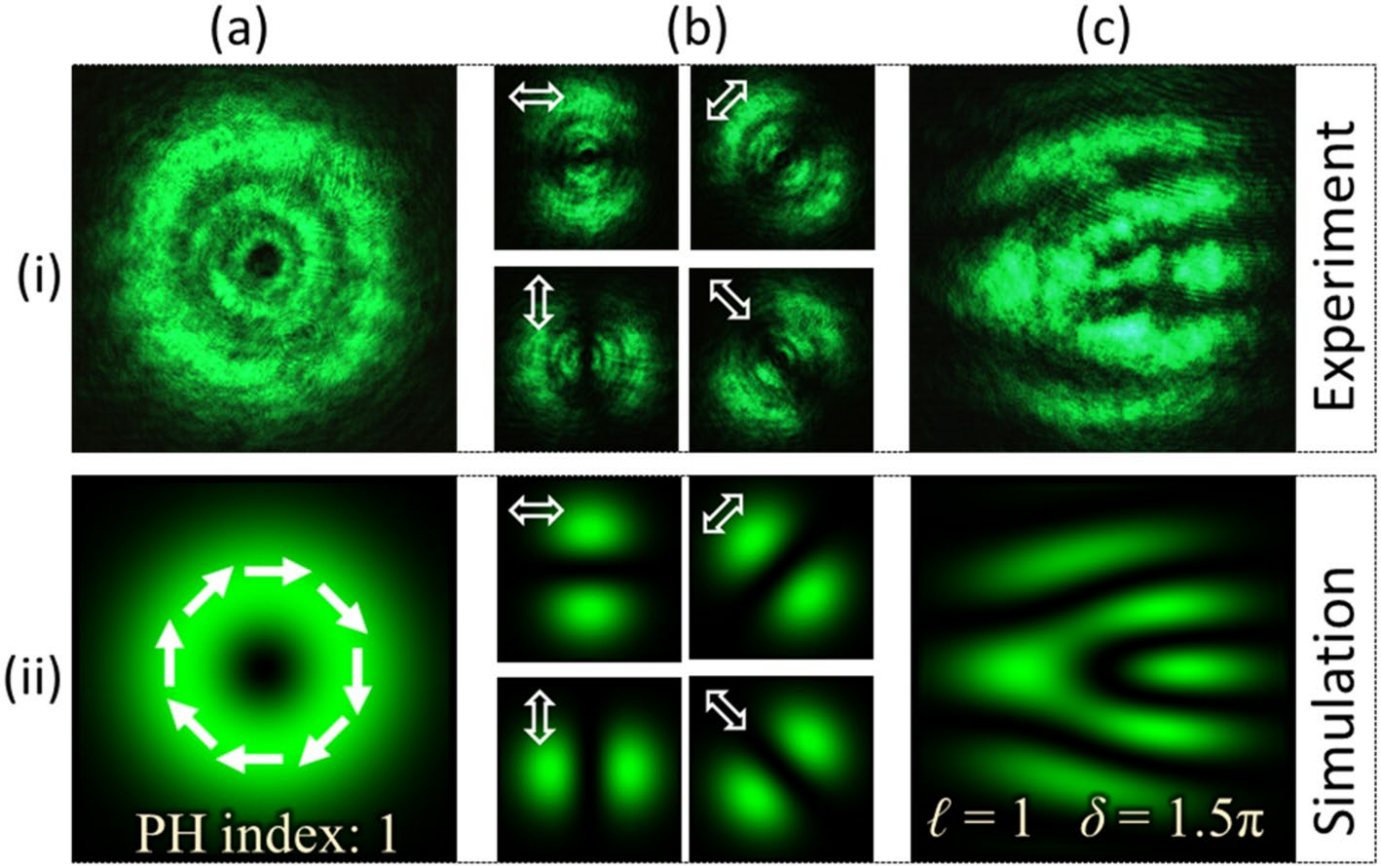
Results for vector-vortex beam of Type-I. (**a**) Intensity, (**b**) intensity after transmitting through a linear polarizer (at four different orientations) and (**c**) interferogram.

PVDs for the Type-I beam are evaluated from Eq. (3) by substituting the parameters *ℓ* = 1 and *δ* = 1.5*π*. The grayscale pattern determined from the PVDs is displayed onto the SLM for beam generation. The captured intensity profile of the generated beam is shown in Fig. 3a–i. The beam has a doughnut-like intensity profile with a dark core at the centre indicating the presence of an optical vortex. It holds a V-point polarization singularity having a Poincaré-Hopf (PH) index of + 1. The beam has spatially-varying polarization which can be verified by analyzing the resultant intensity after transmitting the beam through a linear polarizer. Figures 3b–i show the recorded intensity at four different polarizer’s transmission axis orientations. Here, it is evident that the characterization of VV beams by analyzing their spatial polarization distribution requires the recording and processing of multiple intensity images.

The generated VV beam is directed into the detection set-up shown in Fig. 2b. The optical field formed by the off-axis interference of beam components, as illustrated in Eq. (6), is experimentally obtained and its intensity is recorded as shown in Fig. 3c–i. The recorded interference pattern consists of dark and bright fringes in which a fork-like structure formed by bright fringes is observed. The fork-like pattern appears as a handle connected to three tines which are oriented horizontally. Row (ii) of Fig. 3 presents the simulation results corresponding to results shown in row (i). The simulation has been performed using the MATLAB platform. The fork-like structure is formed because of the defect in the fringes created due to phase singularity in the optical field. A uniform pattern of fringes is observed away from the singularity.

**Figure 4:**
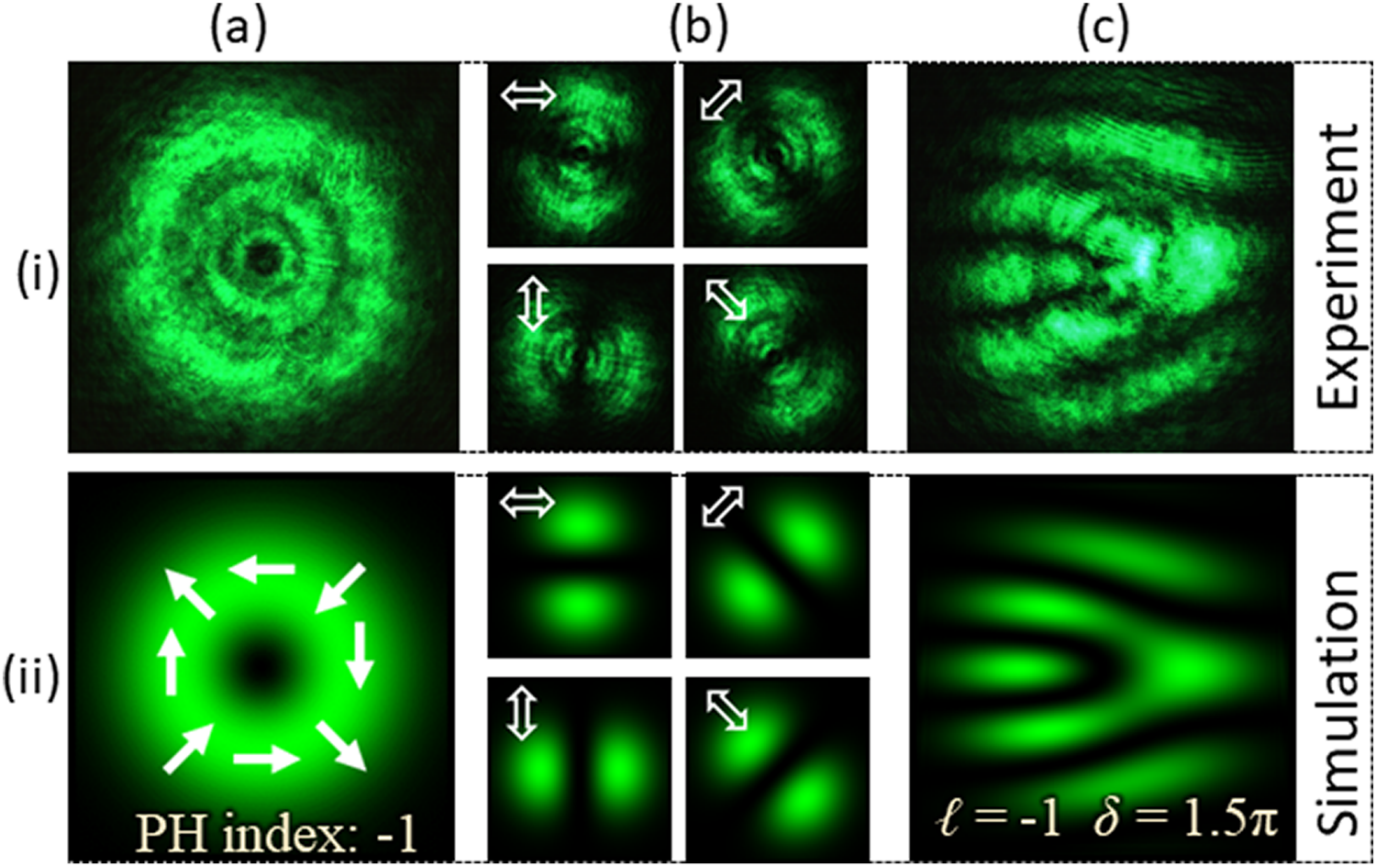
Results for vector-vortex beams of Type-II. (**a**) Intensity, (**b**) intensity after transmitting through a linear polarizer (at four different orientations) and (**c**) interferogram.

Type-II beam is then generated using the same set-up by replacing the grayscale pattern encoded onto the SLM. The parameters of PVDs are *ℓ* = − 1 and *δ* = 1.5π. Type-II beam has a PH index of − 1. The captured intensity of the beam is recorded as shown in Fig. 4a–i. The beam’s intensity distributions captured to verify its polarization are presented in Fig. 4b–i. The interferogram recorded using the detection set-up is shown in Fig. 4c–i. Row (ii) of Fig. 4 shows the corresponding simulation results. Like the Type-I beam; the interferogram consists of a fork-like pattern formed by bright fringes. However, the orientation of the fringe pattern changes and appears as a mirror image of the interferogram formed by the Type-I beam.

**Figure 5:**
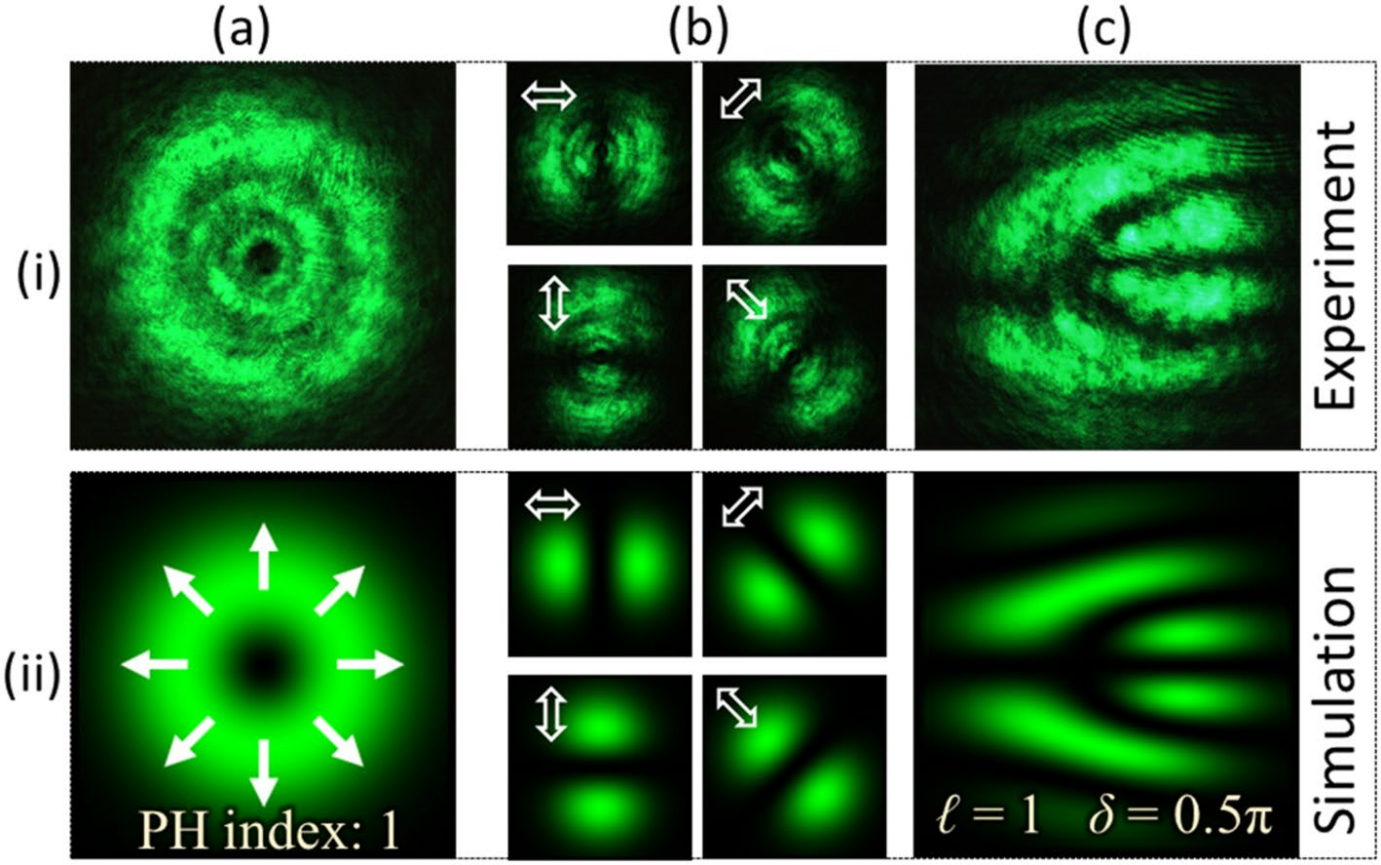
Results for Type-III vector-vortex beam. (**a**) Intensity, (**b**) intensity after transmitting through a linear polarizer (at four different orientations) and (**c**) interference pattern.

The radially polarized beam denoted as Type-III is also generated using the grayscale pattern calculated from Eq. (3) by substituting *ℓ* = 1 and *δ* = 0.5π. Its PH index is + 1. The beam has a doughnut-like intensity profile. The recorded intensity profile is shown in Fig. 5a–i. Figures 5b–i show the beam’s intensity distributions that verify its polarization. The resultant interferogram obtained for detection is shown in Fig. 5c–i. Second row of Fig. 5 shows the corresponding simulation results. The interference pattern for the Type-IV beam consists of a fork-like structure with three tines and a handle formed by dark fringes and oriented horizontally.

**Figure 6:**
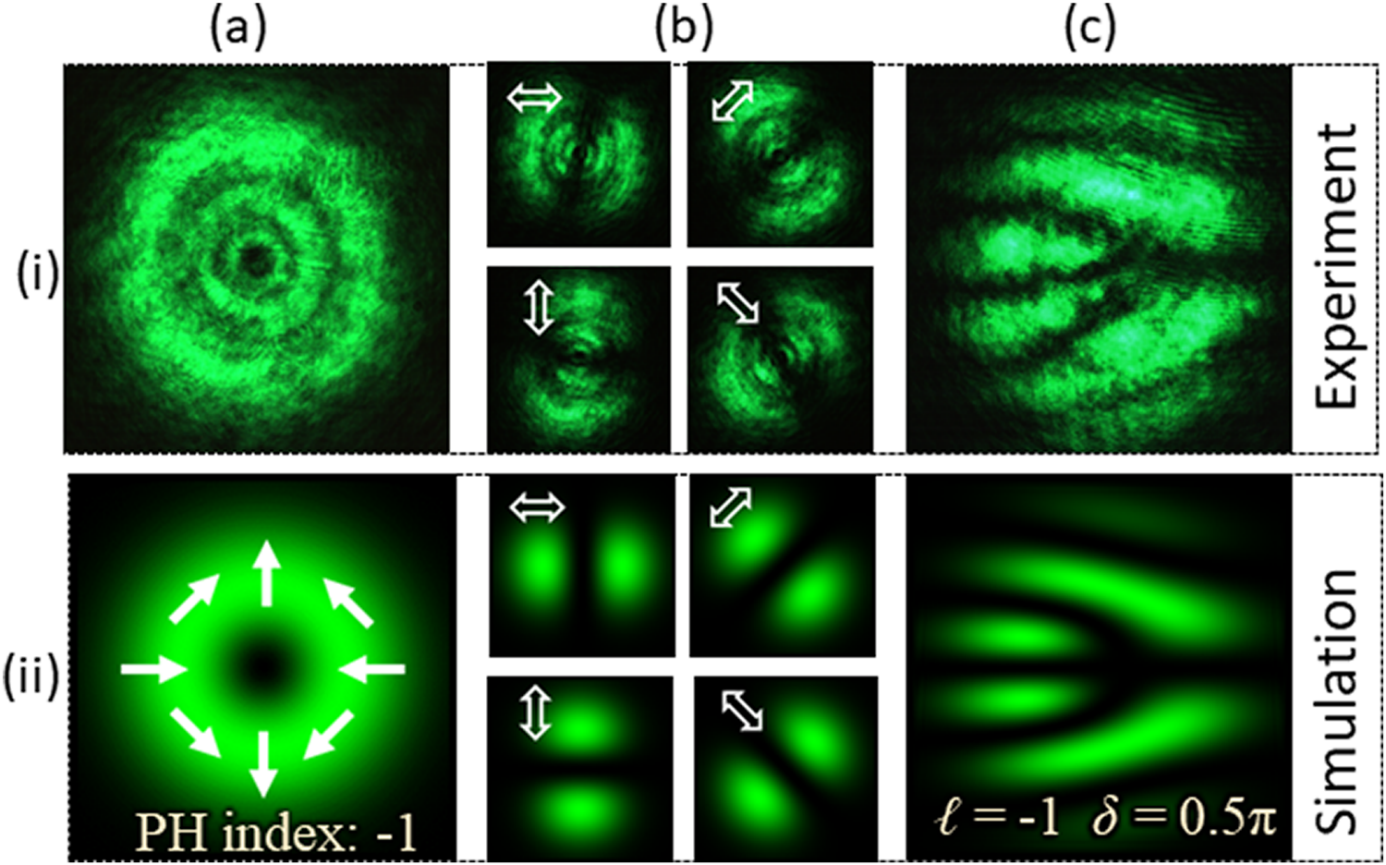
Results for Type-IV vector-vortex beam. (**a**) Intensity, (**b**) intensity after transmitting through a linear polarizer (at four different orientations) and (**c**) interferogram.

Similarly, Type-IV beam is also generated using a grayscale pattern calculated with parameters *ℓ* = − 1 and *δ* = 0.5*π*. Its recorded intensity profile is presented in Fig. 6a–i. The intensity distributions recorded to analyze its polarization are shown in Fig. 6b–i. Finally, the interferogram recorded for its characterization is presented in Fig. 6c–i. Corresponding simulation results are shown in second row of Fig. 6. In the interferogram, the fork-like pattern is formed by dark fringes and the orientation of the fringe pattern is reversed as compared to Type-III.

It can be observed through the results shown in Figs. 2–6 that the experimental results for each beam is in close agreement with the simulation results. These results show that different types of VV beams create different forms of fork-like structures in the interferogram. The magnitude of TC is related to the number of tines in the fork-like structure as $\left| \ell \right| = (n - 1)/2$, where *n* denotes the number of tines formed by either dark or bright fringes. In the interferograms recorded for Types-I, II, III and IV, the number of tines that appeared in the fork structure is three (*n* = 3). Hence, the magnitude of measured TC is one for each beam. Furthermore, the results also show that the opposite sign of TC corresponds to the opposite orientation of the fork structure. In this case, the fork structure is oriented horizontally with tines on the right-hand side for Types-I and III, whereas it is on the left-hand side for Types-II and IV. It shows that Type-I (III) has opposite sign of TC as compared to Type-II (IV).

Based on these analyses, both the sign and magnitude of the TC can be determined for VV beams. Other than TC, the parameter required to identify a VV beam is the phase delay *δ*, which determines the fringe-type that creates fork structure. In this case, the fork structure is formed by a bright fringe for Types-I and II that corresponds to the phase delay value of 1.5*π*. On the other hand, the fork structure is formed by dark fringes for Types-III and IV, which has a phase delay of 0.5*π*. Based on this analysis, both TC and phase delay of an unknown VV beam can be identified from a single interferogram obtained through self-referenced interferometry.

**Figure 7:**
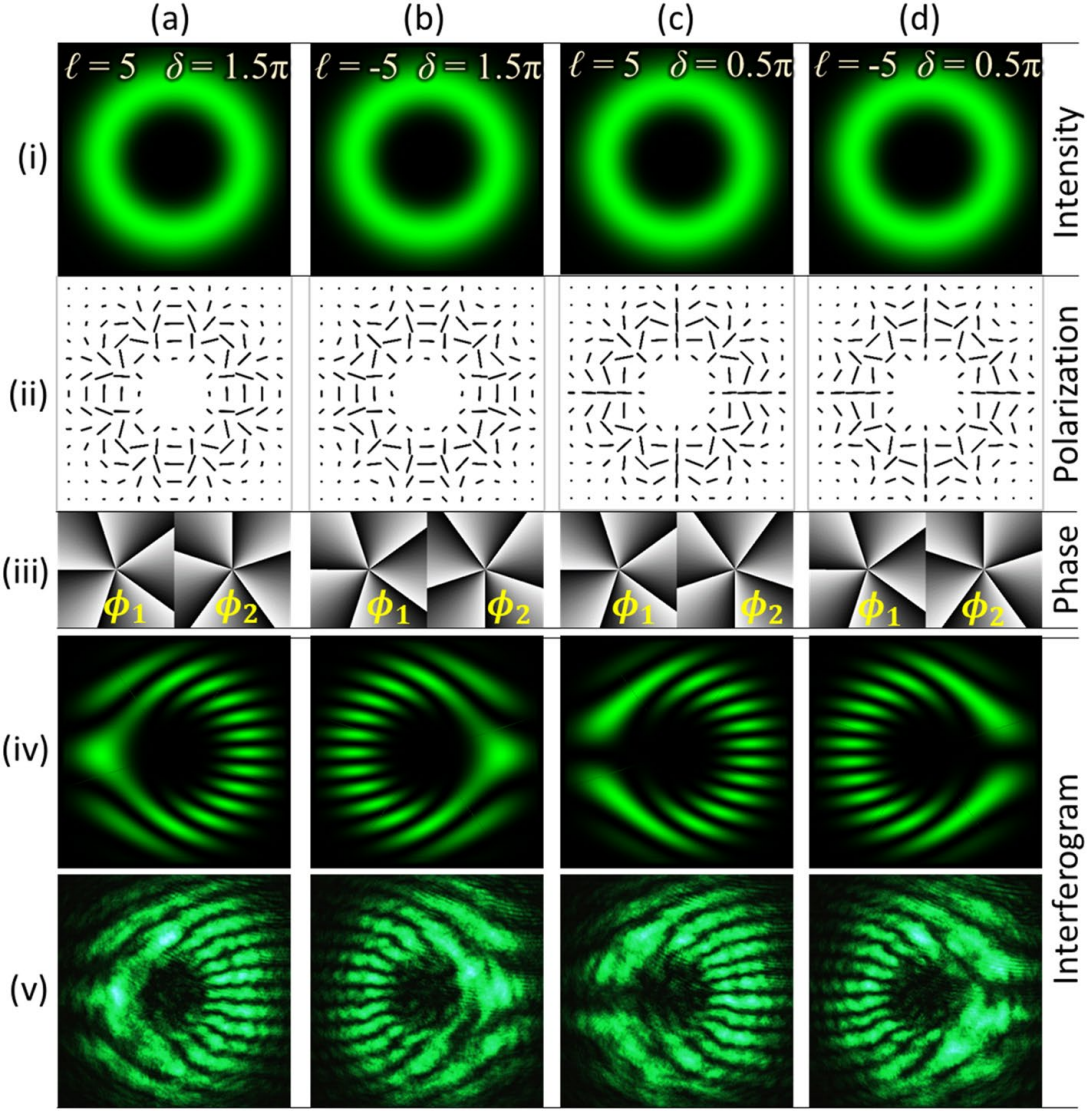
Rows (i–iii) presents intensity, polarization, and phase distributions of high-order vector-vortex beams, respectively. Rows (iv) and (v) show simulated and recorded interferograms, respectively. Columns (**a–d**) correspond to VV beams with |*ℓ|*= 5 similar to Type-I, II, III and IV, respectively.

The experiment has been carried out to verify the feasibility of the proposed method for the detection of high-order VV beams having high values of TC. The experimental procedure as described in previous section is repeated for the generation and detection of VV beams with |*ℓ*|= 5. Similar to the case of Type-I, II, III and IV beams, high-order beams are generated by considering four different combinations of TC (*ℓ*) and the phase delay (*δ*), in Eq. (3). The intensity, polarization, and phase distributions of the resultant VV beams are shown in rows (i–iii) of Fig. 7, respectively. Columns (a–d) of Fig. 7 corresponds to VV beams for different sets of [*ℓ*, *δ*], which are [5, 1.5*π*], [− 5, 1.5*π*], [5, 0.5*π*], and [− 5, 0.5*π*], respectively. Corresponding high-order VV beams are generated and their interferograms are recorded as described in previous sections. Rows (iv) and (v) of Fig. 7 presents the simulated and experimentally captured interferograms, respectively. These results show that as compared to VV beams for |*ℓ*|= 1, the interferograms for high-order VV beams also consist of a fork-like pattern but with a larger number of discontinuous fringes. These extra fringes or the tines reveal the magnitude of TC as $\left| \ell \right| = (n - 1)/2$. For each beam, the number of tines (*n*) is 11 and the magnitude of TC is 5. In the interferograms shown in Figs. 7iv-a and iv-c, tines in the fork structure appeared on the right-hand side, which denotes opposite sign of the input beam’s TC in contrast to the interferograms shown in Figs. 7iv-b and iv-d, where tines appear on the left-hand side. Also, it can be observed that the fork structure in Figs. 7iv-a and iv-b are formed by bright fringe, whereas in Figs. 7iv-c and iv-d, they are formed by dark fringes. This shows the difference of *π* in their phase delay. These observations show that the VV beam having high-order TC can be identified through interference fringe analysis.

**Figure 8:**
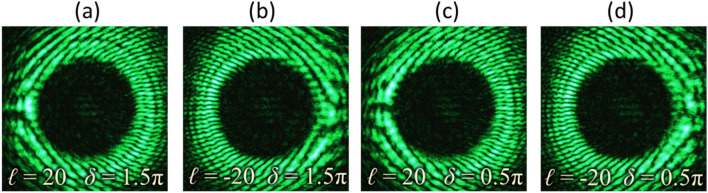
Recorded interferograms for vector-vortex beams for |*ℓ|*= 20. Columns (**a–d**) correspond to different types similar to Type-I, II, III and IV, respectively.

The maximum order of the TC that can be identified would mainly depend on the quality of the resultant interferogram for precise analysis. In the presented set-up, the main factor is the performance of optical devices used for experiment, especially the spatial resolution and dimension of SLM and the camera. An experiment has been performed to observe the interference pattern for VV beams with |*ℓ*|= 20. Figure 8 presents the recorded interferograms, which show the increased number of tines in the fork-shaped fringe pattern. Figures 8a-d correspond to different sets of [ℓ, δ], which are [20, 1.5π], [− 20, 1.5π], [20, 0.5π], and [− 20, 0.5π], respectively. Similar approach of fringe pattern analysis for lower-order VV beams can be applied to reveal the beam’s TC and phase delay. These interferograms verify the formation of a satisfactory fringe pattern for identifying VV beam with |*ℓ*|= 20. In addition to visual identification, analysis of interference fringes can be performed through image processing tools.

**Figure 9:**
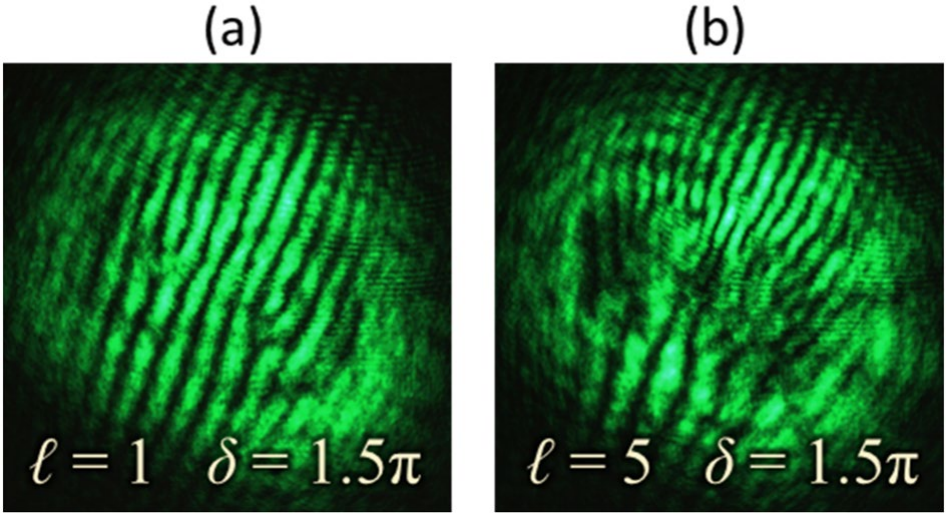
Interferograms recorded when the interferometer is misaligned. (**a**) and (**b**) correspond to Type-I beam for |*ℓ|*= 1 and 5, respectively.

Misalignment during interference may cause unsatisfactory interference pattern because of substantial distortion in the proper fork-like structure. Figure 9 shows the resulting interferograms when the interferometer shown in Fig. 2b is misaligned by altering the orientation of mirror M2. Fringe patterns shown in Fig. 9a,b corresponds to Type-I beam for |*ℓ*|= 1 and 5, respectively. They show that misalignment can cause separation of the beam’s vortices and results in undesirable fringe pattern, especially for higher order beams. Misalignments can also introduce additional phase-shift between the interfering component beams. It can cause inconvenience in analysis or may result in incorrect analysis for the beam characterization. Therefore, it is important to achieve alignment by controlling the tilt and displacement between the beams. A simple method for alignment could be to use a known VV beam to find the position and orientation of the mirror M2 that results in fringe pattern same as the simulated fringe pattern, while the observation plane remains fixed. This step would automatically adjust the path length difference between the two interferometer arms to ensure the desired phase difference between the beam components. This would also reduce the possibility of incorrect beam identification or any ambiguity in deciding the vortex charge. Other factors affecting the formation and shape of fringe patterns may also influence the beam characterization, such as the propagation environment of the beams within the interferometer. The interferogram can be recorded in a vibration isolation environment to avoid undesirable distortions in the fringe pattern.

In a VV beam, both polarization components hold optical vortices with the same magnitude of TC but opposite signs. Figure 9a shows that the information of TCs of the beam components can be observed from the fork-like fringes that appear in the interferogram even with a misaligned set-up. The patterns formed appear similar to the case of interference between two tilted sheared vortex beams. However, the fringe pattern becomes difficult to analyze for higher order beams as the fork-fringes tend to overlap as shown in Fig. 9b. Therefore, the alignment becomes crucial to obtain the desirable fork-like pattern which is formed when the dark core of vortices appearing at the center of each beam component exactly overlaps at the imaging plane.

As compared to the conventional approach to detect VV beam by Stokes parameter measurement, the proposed interferometry method has some significant advantages as well as few limitations. The proposed method is based on single intensity recording of the beam and can be used for rapid detection of vector-vortex beams. No additional change in the experimental set-up or alignment is required for detection of high-order VV beams. Polarization singular points can be characterized into two types; C-points and V-points^49^. The proposed method is suitable for VV beams that hold V-point. They can be characterized using TC (ℓ) and the phase delay (δ). C-point polarization singular beam may also create fork-shaped interference fringe patterns. In such cases, the analysis of fringe patterns to extract the parameters could be modified accordingly. The present method does not reveal the type of polarization singularity directly. But, it can be easily verified by intentionally applying additional lateral displacement. In this case, an input beam with V-point and a C-point could create different patterns. Also, the proposed approach may not directly apply to arbitrary vector beams that don’t hold phase and polarization singularity.

Optical vortices are classified as either scalar or vector, based on the polarization distribution surrounding the singularity point^12,49,62^. Shear interferometry involves interference between the input beam and its wavefront split copy. It can detect scalar vortex beams that hold isolated singularity along their beam axis and are uniformly polarized^63^. It requires analysis of interference fringes having pair of fork-shaped patterns with opposite orientations. The optical field can hold vortices in many other forms. For example, laser beams can hold engineered phase and polarization singularity distributions as composite vortices^64–66^. They can hold multiple singularity points. Shear interferometry can be also applied for their analysis. It usually consists of shear plates made of optical glass and has fixed geometry^63^. It does not allow flexibility in controlling the parameters such as tilt and lateral displacement. Hence, they are not preferred for high-order vortices as the fringe pattern becomes complicated with the increasing order of the azimuthal index. The proposed method involves interference between the polarization components of the input beam. An MZI is required for the implementation as it provides higher flexibility. It is required to control the overlapping between the beam components during interference. In this case, the single fork-shaped pattern is formed, simplifying the analysis even for high-order vector vortices.

## Conclusion

In conclusion, we have proposed a method to detect VV beams based on the principle of self-referenced interferometry. VV beams have been generated, and their detection through interferogram analysis has been demonstrated. To the best of our knowledge, this is the first report that demonstrates the detection of VV beams through analysis of a well-defined single fork-shaped fringe patterns obtained using self-referenced interferometry. The main advantage is that the proposed method requires analysis of a single recorded intensity pattern to obtain the parameters for input VV beam identification. Therefore, this method can be used for rapid detection of VV beams during their applications. Experimental results show that this method is applicable for VV beams even up to azimuthal index, |ℓ|= 20. Hence, it can be applied to characterize high-order VV beams.

## Data Availability

Data underlying the results presented in this paper are not publicly available at this time but may be obtained from the authors upon reasonable request.
